# Cost-effectiveness of vaccination strategies to control future mpox outbreaks in England: a modelling study

**DOI:** 10.1016/j.lanepe.2025.101364

**Published:** 2025-07-01

**Authors:** Xu-Sheng Zhang, Siwaporn Niyomsri, Sema Mandal, Hamish Mohammed, Miranda Mindlin, Bennet Dugbazah, Solomon Adjei, Bukky Owoseni, Andre Charlett, Jessica I’Anson, Elliot Sugars, Merav Kliner, Trish Mannes, Ellie Jewitt, Lorna Gilbert, Samihah Moazam, Claire Dewsnap, David Phillips, Gayatri Amirthalingam, Mary E. Ramsay, Peter Vickerman, Josephine G. Walker

**Affiliations:** a Statistics, Modelling and Economics, Data, Analytics & Surveillance, UK Health Security Agency, London, UK; b Population Health Sciences, University of Bristol, Bristol, UK; c Immunisation and Vaccine Preventable Diseases Division, UK Health Security Agency, London, UK; d Blood Safety, Hepatitis, Sexually Transmitted Infections and HIV Division, UK Health Security Agency, London, UK; e Institute for Global Health, University College London, London, UK; f The National Institute for Health and Care Research Health Protection Research Unit in Blood Borne and Sexually Transmitted Infections at University College London in Partnership with the UK Health Security Agency, London, UK; g Health Protection Operations, UK Health Security Agency, London, UK; h NHS Arden and Greater East Midlands Commissioning Support Unit, Derby, UK; i People Delivery Unit, UK Health Security Agency, London, UK; j Northwest Health Protection Team, UK Health Security Agency, Manchester, UK; k Regional Deputy Director, UKHSA South East, Ashford, Kent, UK; l South London and Maudsley NHS Foundation Trust, UK; m Directorate of Emergency Preparedness, Resilience and Response, UK Health Security Agency, London, UK; n Sheffield Teaching Hospitals NHS Foundation Trust, Sheffield, UK; o Croydon Health Services NHS Trust, Croydon, Surrey, UK; p The National Institute for Health and Care Research Health Protection Research Unit in Behavioural Science and Evaluation at the University of Bristol in Partnership with the UK Health Security Agency, Bristol, UK

**Keywords:** Monkeypox, Vaccination, GBMSM, Mathematical modelling, mpox, Cost-effectiveness

## Abstract

**Background:**

In 2022, a global mpox outbreak occurred among gay and bisexual men who have sex with men (GBMSM). In England, the outbreak was controlled through reductions in sexual risk behaviour and vaccination of high-risk GBMSM. However, mpox continues to circulate, including an expanding outbreak in Africa. We evaluated the most cost-effective vaccination strategy to minimise future mpox outbreaks among GBMSM in England.

**Methods:**

A mathematical model of mpox transmission among GBMSM was developed to estimate the costs per quality-adjusted-life-year (QALY) gained for different vaccination strategies starting in 2024 (10-year time-horizon; 3.5% discount rate; willingness-to-pay threshold £20,000/QALY). Reactive vaccination (only during outbreaks) and/or pre-emptive vaccination (continuous routine) strategies targeting high-risk GBMSM were compared to no vaccination. Baseline projections assumed importation of new mpox cases, and a vaccine effectiveness following 1/2 doses of 78%/89% for 5/10 years at £160/dose. Costs were estimated for case management, vaccination and public health responses during an outbreak.

**Findings:**

All vaccination strategies reduced future outbreaks, gained QALYs and reduced costs compared to no vaccination. Continuous pre-emptive vaccination (daily rate 54 doses) with reactive vaccination (daily rate 81 doses) if there is an outbreak was most cost-effective, saving £8.8 million and gaining 108.6 QALYs over 10-years. Vaccination remains cost-effective if the vaccine costs less than £330/dose. Pre-emptive with reactive vaccination remains the preferred strategy across many sensitivity analyses, with just pre-emptive vaccination at a higher rate becoming the preferred strategy in some sensitivity analyses. Just reactive vaccination only becomes the preferred strategy when public health response costs are not included, and in this case the vaccine has to cost less than £110 per dose for vaccination to be cost-effective.

**Interpretation:**

Vaccination of high-risk GBMSM is likely to be a cost-saving strategy for preventing future mpox outbreaks.

**Funding:**

10.13039/501100000272NIHR and 10.13039/100010269Wellcome Trust.

**Research in context**

**Evidence before this study**

On going transmission of mpox in Africa and continued importations of mpox to England have raised concerns that new mpox outbreaks could occur among gay and bisexual men who have sex with men (GBMSM) in England especially if levels of vaccine-induced protection reduce over time. We searched PubMed, bioRxiv and medRxiv for articles published from beginning May 2022 to 28 June 2024 with the following keywords: ((“monkeypox” OR “mpox” OR “mpx”) AND (“model” OR “modelling” OR “modeling”) AND (“vaccine” OR “vaccination” OR “cost-effectiveness” OR “cost-effective”)). The search identified numerous articles involving transmission modelling that assessed the impact of interventions on mpox transmission, but only eight considered what is needed to prevent future outbreaks and none evaluated the cost-effectiveness of vaccination. Existing model analyses have shown that future outbreaks may be controlled by vaccinating close contacts of cases and individuals in large sexual networks, as well as pre-emptively vaccinating high-risk individuals before outbreaks occur. However, none of these analyses undertook detailed site-specific modelling. Other more detailed modelling for specific settings (US, Canada, Netherlands and England) have shown that existing levels of vaccination following the 2022 outbreak may have reduced future outbreaks, including potential clade I mpox outbreaks, but these analyses did not model future vaccination strategies. The only economic analysis for mpox compared the costs of vaccination to not vaccinating for the general population in Jeddah, Saudi Arabia. Unfortunately, this economic analysis used implausible data (respiratory infection contact rates) to simulate the transmission of mpox and used little data on the health-related costs of mpox disease.

**Added value of this study**

Combining a previously validated model of mpox infection among GBMSM in England with real data on the costs of care for mpox, vaccination and public health responses, we evaluated the most cost-effective vaccination strategy to prevent future mpox outbreaks. We modelled reactive (only vaccinate during outbreaks) and pre-emptive (routine vaccination irrespective of outbreaks) vaccination strategies targeting high-risk GBMSM. Our analyses show that vaccination is likely to be cost-saving compared to not vaccinating, with continuous pre-emptive vaccination (daily rate 54 doses) combined with reactive vaccination (daily rate 81 doses) if there is an outbreak being the most cost-effective strategy. Although a combined strategy is optimal over many sensitivity analyses, just pre-emptive vaccination at a higher rate is sometimes a more cost-effective strategy, while just reactive vaccination is preferred if public health response costs are not included.

**Implications of all the available evidence**

Expanding outbreaks of mpox in Africa and the ongoing importation of mpox cases in non-endemic countries means that countries need to be prepared for future mpox outbreaks. Our analyses give robust evidence that continued mpox vaccination is a cost-saving strategy for minimising the likelihood of future mpox outbreaks among GBMSM in England and comparable countries. These findings have been used as evidence to recommend for a vaccination programme among high-risk GBMSM in England. Other countries should consider similar strategies to prevent future outbreaks among GBMSM.

## Introduction

In May 2022, a global outbreak of mpox (clade IIb) occurred that primarily affected gay, bisexual, and other men who have sex with men (GBMSM).^1^ In response, many nations implemented measures to tackle the outbreaks, including provision of vaccines and public health measures.^2^ The global outbreak declined after July 2022, with there being 91,788 confirmed cases from 116 countries by October 2023.^3^ In England, cases peaked in mid-July and then declined, with 3412 reported cases by 16 September 2022.^4^

The Modified Vaccinia Ankara (MVA) vaccine, originally developed for smallpox, was rolled out in England and other countries during the 2022 outbreak, with subsequent studies estimating that the vaccine provided ∼85% efficacy against mpox.^5^, ^6^, ^7^ Although immunity can persist for decades following live smallpox vaccination,^8^ the duration of protection for the non-replicating MVA vaccine is uncertain. Model analyses suggest that the vaccines delivered in England had limited impact on the mpox outbreak due to their late roll-out (late June 2022 onwards). However, they may have prevented subsequent outbreaks^9^^,^^10^ due to levels of vaccination among eligible GBMSM (69%)^11^ being above the herd immunity threshold projected by previous modelling (48% among high-risk GBMSM).^10^

A low incidence of mpox (clade IIb) has continued in England during 2023 (137 reported cases).^6^ An expanding outbreak is also ongoing in sub-Saharan Africa, with a new clade (Ib) showing evidence of sustained transmission.^12^ This resulted in WHO declaring mpox a Public Health Emergency of International Concern (PHEIC) in August 2024,^13^ raising concern that future outbreaks could occur especially if population immunity levels decrease. We used modelling to determine the most cost-effective vaccination strategy for minimising future mpox outbreaks among GBMSM in England.

## Methods

We adapted our existing deterministic compartmental model of mpox transmission among GBMSM in England^10^ to simulate the cost-effectiveness of different vaccination strategies over 10-years from 2024. The model was parameterised and calibrated using surveillance data from the 2022 outbreak, two GBMSM behavioural surveys undertaken in 2021 and 2022,^11^^,^^14^ and data on importation of mpox cases.^6^ The cost per quality-adjusted-life-year (QALY) gained was used to compare various vaccination strategies targeting GBMSM at high-risk of mpox for preventing future outbreaks. A health services perspective was used for estimating costs.

### Model structure

We initiated our mpox model^10^ in April 2022 with a fully susceptible GBMSM population and a rate of imported mpox cases (Poisson process). Susceptible individuals become latently infected through sexual exposure to mpox cases. They then enter the infectious period, which is stratified into mild, moderate, and severe disease. Individuals then recover and develop immunity or are diagnosed and isolated, defining the effective infectious period. The model is stratified by vaccination status: none, first and second dose. The model also incorporates recruitment of new GBMSM and waning immunity following vaccination and post-infection. Waning immunity results in individuals returning to a susceptible class where they can be reinfected but are not eligible for revaccination. Revaccination was not modelled because current vaccine policy in England does not allow reinforcing or booster doses, and no vaccination post infection occurs due to supply constraints. The model does not include transmission of mpox to other population groups.

The model divides the England GBMSM population (769,000^15^) into four groups at low- and high-risk of mpox transmission and whether they attend sexual health services (SHS) or not. These strata are included because most mpox cases occurred among high-risk GBMSM in 2022^10^ and vaccination was targeted to these GBMSM attending SHS.

**Fig. 1:**
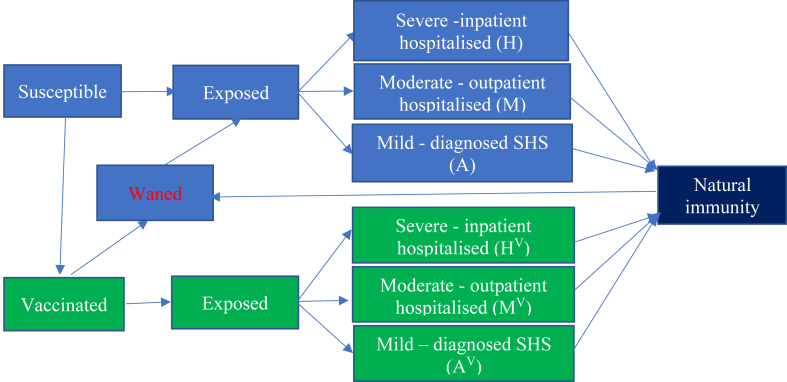
Schematic of the mpox transmission model among GBMSM for assessing the impact and cost-effectiveness of different future vaccination scenarios. Stratifications by low and high risk for mpox infection and attendance at sexual health services are not shown, and vaccination is only shown as one stratification although there are stratifications for 1 or 2 doses of vaccine in the model.

The three levels of disease severity capture differences in health care costs and quality-of-life. Based on observed cases in England,^16^ severe disease cases were defined as those having inpatient hospital care, while moderate cases required outpatient hospital care and mild cases had no hospital care but attended SHS. During the 2022 outbreak, the UK reported no mpox-related deaths,^4^ and so this was not modelled. Fig. 1 gives a simplified model schematic, with a full model description in Appendix pp 4–14.

### Model parameterisation and calibration

The model was parameterised and calibrated to the 2022 outbreak^10^ and then run till end-2023, after which we modelled various vaccination scenarios from 2024. Data used for this calibration are described elsewhere^10^ and briefly here, with more details in Table 1 and Appendix pp 8–12.

**Table 1:** Posterior estimates of transmission parameters for model.

Model parameter	Median (95%CrI)	Reference
Transmission coefficient	0.56 (0.44,0.71)	^10^
Timepoint of outbreak (days) after which contact rate and infectious period decrease	50.56 (40.28,68.51)	
Relative reduction in contact rate after timepoint in outbreak	0.45 (0.25,0.56)	
Effective infectious period (days) at start of outbreak	3.02 (2.54,3.93)	
Effective infectious period (days) after timepoint in outbreak	2.38 (1.98,3.31)	
Parameter defining the rate of change in behaviour and effective infectious period after timepoint in outbreak (Appendix pp 8–9)	0.016 (0.011,0.039)	
Effectiveness of 1st dose of vaccine	0.78 (0.54–0.89)	^5^^,^^6^
Effectiveness of 2nd dose of vaccine	0.89 (0.78–0.99)	^17^
Effectiveness of natural infection for protecting against re-infections	1.00	Assumption
Duration of protection due to 1st dose of vaccine	5.0 years	Assumption
Duration of protection due to 2nd dose of vaccine	10 years	Assumption
Duration of protection due to natural infection	10 years	Assumption
Mean rate of new infections entering population per month from 17th November 2022 onwards	6	^6^
Criteria for start of outbreak response	120 cases in 3 months	Assumption
Criteria for end of outbreak response	60 cases in 3 months	Assumption
Proportion of GBMSM that are high-risk for mpox	11.9%	^14^
Mean contact rate for low and high risk GBMSM in last 4 months	1.6 (low), 22.3 (high)	^14^
Proportion of low- and high-risk GBMSM that attend sexual health services (SHS)	36.8% (low), 69.3% (high)	^14^

Until 17 November 2022, data on actual imported cases were used in the model.^10^ Following this, we assumed an average importation rate (∼6 cases/month) based on the estimated number of imported mpox cases in England in 2023 (n = 73).^6^ These cases were assumed to have the same disease attributes as the clade IIb infections in the 2022 outbreak.

Risk status was defined based on whether GBMSM have less than 10 or more than 10 anal sex partners in the last four months, with the proportion in these categories and attending SHS being estimated from the 2021 Reducing Inequalities In Sexual Health (RiiSH) study, an internet-based survey of 1039 GBMSM in England.^14^ Because of a lack of UK data suggesting that GBMSM change sexual risk levels and because the RiiSH study found a stable proportion of high-risk GBMSM over age (Appendix pp 4), we did not stratify the model by age and assumed GBMSM do not transition between risk strata. As in our previous analysis,^10^ a reduction in sexual risk behaviour was assumed during the 2022 outbreak (and any subsequent outbreak) to reproduce the downturn in cases.

From mid-2022 to end-2023, vaccines were assigned in the model among high-risk GBMSM based on weekly numbers of first and second doses.^18^ We assumed a vaccine effectiveness against mpox infection of 78% (95%CI: 54–89%)^5^^,^^6^ for one dose and 89% (95% CI: 78–99%)^17^ for two doses. Based on immunological principles and other similar vaccines, we expect vaccine protection to last greater than 5 years.^19^ We therefore assumed a duration of protection of 5 and 10 years for one and two doses, respectively. The duration of protection induced by natural infection was assumed to be equivalent to receiving two vaccine doses, with 100% effectiveness against infection.

Bayesian Markov chain Monte Carlo sampling was used to calibrate the model to case data among males in England from April to 12 August 2022, with model fits being validated against case data to 16 November 2022.^10^ Model fits were used to project the impact of vaccination strategies from 2024, with the range across model fits being used to estimate the mean and 95% credibility interval (95%CrI) for all projections.

### Health utilities

We estimated QALY weights for GBMSM without mpox using health-related quality-of-life (HRQoL) data collected in the RiiSH-MPOX survey undertaken in December 2022.^11^ As no QALY estimates were available for mpox, we assumed disutilities for mpox based on disability weights for mild (0.006 (0.002–0.012)), moderate (0.051 (0.032–0.074)), and severe (0.133 (0.088–0.190)) infectious disease from the Global Burden Diseases 2021 study.^20^ Disability weights were assumed to be the same as disutilities for calculating QALYs and subtracted from the baseline HRQoL data to estimate the utility weights for mpox (Table 2). Additionally, HRQoL utility weights from a UK study of herpes zoster (presumed similar pain levels to mpox)^28^ were used to expand the uncertainty bounds. Further details in Appendix pp 21.

**Table 2:** Cost and utility parameters. All costs are presented in 2022 British Pounds (£).

Parameters	Base case value (lower–upper bound)^a^	Source/Comments
**Unit costs**		
Clinical case management costs		
Mild cases	£378.63 (£303.22–£454.04)	^16^^,^^21^, ^22^, ^23^ and Clinical experts. Patients with mild symptoms were assumed to be diagnosed and treated at sexual health services (SHS) and isolated at home. Mild symptoms typically presented with few lesions. All cases were confirmed with PCR by Rare and Imported Pathogens Laboratory (RIPL).
Moderate cases	£730.67 (£577.00–£730.67)	^21^^,^^24^^,^^25^Patients with moderate level of symptoms were defined as those who attended hospital through the emergency department (ED) and were not admitted. An estimated 14.4% of these patients had attended SHS before visiting ED.
Severe cases	£3991.22 (£3776.00–£3991.22)	^21^^,^^24^, ^25^, ^26^Patients with severe mpox symptoms were defined as those who received inpatient hospital care. Based on analysis of Secondary Uses Service (SUS) and the emergency care data set (ECDS), 63.4% of patients admitted to hospital for mpox first attended ED prior to admission. We assumed the remainder (36.6%) attended SHS prior to hospital admission and ∼4% of hospitalized patients required intensive care
Vaccination administration costs		
First dose administration	£36.85 (fixed)	SHS tariff for mpox.^21^ Costs include staff time costs for registration, consultation, vaccine administration, and health promotion, as well as consumables such as gloves, plasters and cotton
Second dose administration	£25.78 (fixed)	SHS tariff for mpox.^21^ Costs assume a reduced consultation time compared to initial visit
Vaccine cost	£160.00 (fixed)	^27^Assumed cost of Shingrix vaccine.^b^
Public health response costs		
Weekly outbreak-related overhead costs	£81,290 (£73,714–£88,866)	Public health response costs were gathered from: i) the UKHSA regional teams where most Mpox cases occurred in 2022, ii) the central UKHSA National Response Centre, and iii) cross-UKHSA Enhanced incident costs from the UKHSA Directorate of Emergency Preparedness, Resilience and Response. This data was used to estimate the cost of a national response (for England) including both regional and central response activity.
Per case costs	£416 (£184–£779)	Per case cost covers the cost associated with staffing the mpox cell in London and across England.
**Utilities**		
Baseline utility	0.839 (fixed)	^11^
Mild Mpox	0.833 (0.827–0.837)	^20^^,^^28^
Moderate Mpox	0.788 (0.765–0.829)	^20^^,^^28^
Severe Mpox	0.706 (0.649–0.821)	^20^^,^^28^

a All uncertainty ranges were sampled using triangle distribution.

b The cost of the mpox vaccine is confidential, so we used the cost for the Shingrix vaccine (£160 per dose^27^; thought to have a comparable cost) in the baseline analysis, and estimated the maximum threshold vaccine cost for each intervention to be cost-effective.

### Costs

A health services perspective was used for estimating costs (2022 British pounds £) using data from the 2022 outbreak. Costs for clinical case management (for different disease severity levels), public health responses during an outbreak and administering the vaccine were included, using data from various sources and incorporating uncertainty. Costs for public health response measures undertaken during the 2022 outbreak include costs related to each case (e.g., contact tracing) and outbreak-related overhead costs. Details of these costs are given in Table 2, with more details in Appendix pp 12–21.

### Future vaccination scenarios

From January 2024, we modelled the costs and impact of the following vaccination scenarios for high-risk GBMSM attending SHS (Appendix pp 9–11):

• Counterfactual scenario: No vaccination from 2024 onwards, with vaccination in 2022–2023.
• Pre-emptive vaccination irrespective of outbreak: Vaccines given continuously at constant rates of 13, 27, 41, 54, 81, and 135 per day. These rates were chosen because they achieve a vaccination coverage of 5, 10, 15, 20, 30, and 50% in a year, respectively, if initiated from scratch.
• Reactive vaccination when outbreak triggered: Vaccines given at constant rate once an outbreak response is triggered and stopped when outbreak ends. We consider the rate achieved in the 2022 outbreak, 465 per day, and lower rates similar to pre-emptive vaccination.

We also modelled combined scenarios including both pre-emptive and reactive vaccination. For reactive vaccination, our baseline analysis assumes that an outbreak response is triggered if there are more than 120 cases in 3 months and ends when there are less than 60 cases in 3 months. We assume that a public health response occurs during an outbreak response, and that the same reduction in sexual risk behaviour occurs as in the 2022 outbreak.^10^ This risk reduction is not sustained after each outbreak, as suggested by rebounds in STI trends following the 2022 outbreak.^29^ In all scenarios, we assume first vaccine doses are distributed among susceptible individuals and second doses are administered to those who have not yet received them, with 56% of individuals receiving a second dose.^18^ However, if no more high-risk GBMSM require a first dose, then all vaccinations are provided as second doses. Fractional doses are considered as a sensitivity analysis.

### Cost-effectiveness analysis

We evaluated the incremental cost-effectiveness ratio (ICER) in terms of the incremental cost per QALY gained for each vaccination scenario over a 10-year period from January 2024. ICERs were estimated by comparing the estimated costs and QALYs for each vaccination scenario to the counterfactual (no vaccination) scenario. A full incremental cost-effectiveness analysis was also conducted to compare between scenarios and so determine the most cost-effective vaccination scenario. This involved ordering the vaccination scenarios in terms of increasing cost, and calculating incremental costs and QALY gains, eliminating any scenario which are dominated (higher cost and fewer benefits) or extendedly dominated (higher ICER and fewer benefits). In addition, the incremental net monetary benefit (INMB) was calculated for each scenario (compared to no vaccination), calculated as incremental QALYs multiplied by the willingness-to-pay (WTP) threshold minus the incremental costs for that intervention scenario. The highest INMB represents the most cost-effective scenario at a particular WTP threshold, with any INMB greater than 0 being cost-effective at that threshold. We did not calculate the benefit-cost ratio as discussed in Appendix pp 14.

For all comparisons, probabilistic sensitivity analyses were undertaken that sampled model, utility and cost parameters 500 times to produce uncertainty bounds around the incremental costs and QALYs. Costs and QALYs were discounted at 3.5% per year and WTP thresholds of £20,000 or £30,000 per QALY were used.^30^ We also estimated the maximum threshold vaccine price for vaccination to be cost-effective defined as 50% of probabilistic ICER estimates below the £20,000/QALY threshold or 90% below £30,000/QALY.^30^

### Sensitivity analyses

Because of numerous uncertainties, we undertook nineteen ‘one-way’ sensitivity analyses to evaluate whether altering specific parameter estimates or model assumptions changed which vaccination strategy was most cost-effective. As described in Table 3 and Appendix pp 22–27, sensitivity analyses included changes in the duration of vaccine protection, case importation rates, outbreak response criteria, time horizon, allowing vaccination of GBMSM with waned protection, and excluding public health response costs. For each sensitivity analysis, all other parameters and assumptions remained the same as in the baseline analysis.

**Table 3:** Sensitivity analyses to evaluate whether specific changes in our baseline assumptions change which vaccination strategy is most cost-effective.

Sensitivity analysis	Optimal vaccination scenario	Total number of infections	Incremental costs compared to no vaccination (1000s £)	Incremental QALYs compared to no vaccination
**Baseline**	PV54 + RV81	1167 (422, 2131)	−8825 (−12,340, −5528)	108.64 (60.8, 168.55)
Annual discount rate of 1.5% instead of 3.5%;	PV81	1160 (440, 2091)	−10,721 (−14,515, −6935)	124.62 (69.85, 193.34)
Only include direct health care costs so remove public health response costs from baseline;^a^	RV27^a^	2994 (1925, 4291)	3714 (2502, 4688)	90.31 (46.02, 141.76)
Societal perspective: Include productivity losses due to mpox disease, estimated using data from the RiiSH-MPOX survey^11^ and UK data on employment and average salaries (Appendix pp 20–21);	PV81	1160 (440, 2091)	−21,127 (−27,803, −14,070)	109.06 (61.15, 169.35)
Long duration of vaccine protection of 10 and 20 years for one and two doses, respectively, instead of 5 and 10 years;	PV13 + RV204	234 (0, 1123)	−2690 (−6036, 436)	23.4 (9.9, 47.56)
Short duration of vaccine protection of 2.5 and 5.0 years for one and two doses, respectively;	PV135	17,317 (8742, 28,029)	−14,320 (−22,530, 1169)	287.53 (51.71, 431.46)
Low rate of 1 imported case per month instead of 6;	PV27 + RV204	460 (39, 1120)	−8004 (−12,041, −3944)	100.57 (51.95, 160.83)
High rate of 10 imported cases per month;	PV271	2125 (1089, 3379)	−6474 (−11,427, −1951)	93.37 (31.58, 156.62)
Low outbreak response criteria of 96 cases in 3 months (baseline 120), which ends when there are less than 48 cases in 3 months (baseline 60);	PV271	1209 (572, 2044)	−6818 (−10,093, −3841)	89.55 (47.84, 139.20)
High outbreak response criteria of 144 cases in 3 months, which ends when there are less than 72 cases in 3 months;	PV41 + RV81	1176 (365, 2153)	−10,324 (−13,995, −6309)	123.36 (69.92, 188.07)
No reductions in risk behaviour during outbreaks; baseline assumes decrease occurs during each outbreak;	PV54 + RV81	48,015 (3326, 103,020)	−103,136 (−138,799, −71,351)	1524.57 (850.68, 2251.8)
Alternative definition of low- and high-risk GBMSM using data on rates of physical contact from the RiiSH-MPOX survey^11^^;^^a^	PV41 + RV204^a^	448 (297, 741)	2354 (−241, 5215)	45.46 (24.71, 81.89)
Assume breakthrough infections have half the chance of experiencing moderate and severe mpox disease than in baseline scenario;	PV54 + RV81	1167 (422, 2131)	−8806 (−12,316, −5515)	107.91 (60.44, 167.05)
Half of vaccinations are delivered as fractional (1/4) doses with 25% lower effectiveness following 1 and 2 doses.	PV271	1391 (645, 2484)	−13,270 (−16,603, −9664)	106.69 (59.79, 164.49)
Greater percentage (+20 percentage points) of GBMSM attend Sexual Health Services	PV54 + RV81	876 (182, 1668)	−6733 (−10,031, −3173)	110.37 (61.64, 168.63)
On average, all high-risk GBMSM transition to being low-risk GBMSM before they age out of the model (transition rate 2.9% per year) with equivalent number moving to high-risk, instead of no movement between risk classes	PV41 + RV135	954 (314, 1755)	−9448 (−13,849, −5687)	164.57 (93.75, 247.36)
Increase time horizon from 10 years to 20 years	PV41 + RV81	13,222 (7984, 18,772)	−25,538 (−34,174, −18,348)	386.33 (227.93, 544.23)
GBMSM with waned protection (either vaccine induced or from natural infection) are immediately eligible for re-vaccination	PV27 + RV81	281 (50, 589)	−7220 (−10,721, −4134)	113.23 (61.71, 178.21)
Distribution of time GBMSM remain within modelled population changed from exponential to gamma	PV81	1150 (431, 2072)	−9433 (−12,932, −5873)	113.43 (63.88, 175.37)
Reduced infectious period during outbreak stays after end of outbreak	PV41 + RV204	630 (0, 1715)	−9256 (−13,982, −4989)	111.57 (61.85, 167.08)

Sensitivity analyses include the following changes which are described in detail in Appendix pp 22–27 and Appendix Tables S15–S35, with all other parameters and assumptions remaining the same as in the baseline analysis.

a Signifies that these sensitivity analyses are not cost-effective unless the vaccine price is lower than the baseline price. When public health response costs are not included, the vaccine needs to be less than £110 per dose for this vaccination scenario to be cost-effective, while when we use an alternative definition for low- and high-risk GBMSM, the vaccine needs to be less than £145 per dose for this vaccination scenario to be cost-effective.

### Role of the funding source

The study sponsors (NIHR) had no involvement in the study design; in the collection, analysis, and interpretation of data; in the writing of the report; and in the decision to submit the paper for publication.

## Results

### Baseline impact projections

**Fig. 2:**
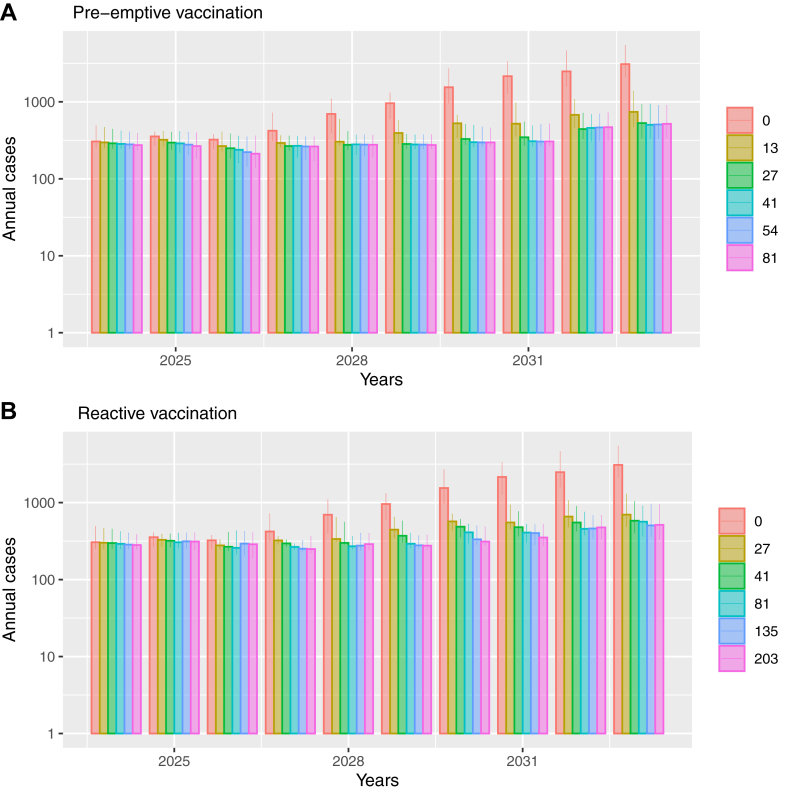
Annual cases over next 10 years (log scale) under different pre-emptive (A) and reactive (B) vaccination scenarios. Modelled vaccination rates are 13, 27, 41, 54 and 81 doses per day for pre-emptive vaccination (A) and 27, 41, 81, 135 and 203 doses per day for reactive vaccination (B). The error bars represent 95% CrI.

With no vaccination from 2024 (counterfactual scenario), the baseline model projects outbreaks will occur approximately yearly (Appendix Fig. S3), with the median number of yearly cases increasing from 306 (95%CrI 253–492) in 2024 to 3089 (95%CrI 2137–5431) in 2033 (Fig. 2) and 11,384 (95%CrI: 7299–15,770) cases occurring over 2024–2033 (Table 4). Importantly, these outbreaks are dependent on imported infections, with the model projecting that mpox cases will reduce to very low levels (less than 0.01) after 2024 without imported infections (Appendix Fig. S5). Outbreaks also reduce with vaccination. For instance, with pre-emptive vaccination strategies that deliver greater than or equal to 41 vaccine doses per day, the yearly number of cases remains below 308 up to 2031 (including imported infections; Fig. 2). Conversely, the number of yearly cases remains below 510 with reactive vaccination strategies that deliver greater than or equal to 135 vaccine doses per day during each outbreak. Projections (Appendix Fig. S3) suggest that these levels of vaccination keep population-level immunity above the herd immunity threshold (48.3%^10^) among high-risk GBMSM until about 2031.

**Table 4:** Epidemic characteristics, costs and cost-effectiveness projections for different vaccination programmes over 2024–2033 undertaken among GBMSM at high risk for mpox who attend sexual health services.

Vaccination scenario	Total number of infections	Outbreak duration (days)	Total Cost (1000s £)	Total QALYs	Incremental costs compared to no vaccination (1000s £)	Incremental QALY compared to no vaccination	ICER compared to no vaccination	Fully incremental analysis	INMB compared to no Vaccination (£20,000/QALY)	INMB compared to no vaccination (£30,000/QALY)
No Vaccination counterfactual	11,384 (7299, 15,770)	2298 (1871, 2520)	32,826 (25,194, 39,213)	5,556,871 (5,556,794, 5,556,936)	–	–	–	Dominated	–	–
Pre-emptive vaccination (PV) but no reactive vaccination										
PV 41 vaccines per day	1220 (442, 2215)	1046 (512, 1562)	24,213 (18,316, 30,224)	5,556,979 (5,556,961, 5,556,997)	−8613 (−11,953, −5794)	107.92 (60, 167.62)	Cost-saving	Dominated	10,771,262 (7,618,678, 14,335,450)	11,850,459 (8,310,640, 15,684,403)
PV 54 vaccines per day	1171 (422, 2156)	989 (492, 1533)	24,021 (18,422, 30,254)	5,556,980 (5,556,960, 5,556,997)	−8805 (−11,991, −5578)	108.6 (60.64, 169.71)	Cost-saving	Dominated	10,977,081 (7,423,863, 14,877,387)	12,063,102 (8,098,608, 16,302,538)
PV 81 vaccines per day	1160 (440, 2091)	956 (473, 1448)	24,016 (18,724, 29,701)	5,556,980 (5,556,959, 5,556,997)	−8809 (−12,197, −5341)	109.06 (61.15, 169.35)	Cost-saving	£38,095/QALY compared to PV54RV81	10,990,635 (7,042,115, 15,106,175)	12,081,261 (7,733,561, 16,440,024)
Reactive vaccination (RV) but no pre-emptive vaccination										
RV 41 vaccines per day	2324 (1488, 3358)	1647 (1317, 1907)	27,965 (22,682, 32,370)	5,556,969 (5,556,946, 5,556,990)	−4861 (−7691, −2051)	97.56 (49.47, 154.15)	Cost-saving	Dominated	6,811,683 (3,335,332, 10,380,132)	7,787,247 (3,934,588, 11,841,973)
RV 81 vaccines per day	1710 (1092, 2438)	1413 (1096, 1669)	26,516 (21,320, 30,646)	5,556,975 (5,556,956, 5,556,993)	−6310 (−9398, −3220)	103.94 (53.58, 165.22)	Cost-saving	Dominated	8,388,479 (4,448,684, 12,277,012)	9,427,847 (5,157,204, 13,876,560)
RV 135 vaccines per day	1490 (908, 2257)	1279 (955, 1563)	25,717 (20,203, 30,489)	5,556,977 (5,556,959, 5,556,994)	−7109 (−10,476, −3759)	105.31 (54.32, 168.36)	Cost-saving	Dominated	9,215,193 (5,078,558, 13,288,526)	10,268,282 (5,662,918, 14,665,559)
Pre-emptive vaccination (PV) plus reactive vaccination (RV) at 81 doses per day when outbreak is triggered, otherwise at rate given below										
PV 27 vaccines per day	1271 (559, 2189)	1089 (607, 1551)	24,374 (18,207, 30,244)	5,556,978 (5,556,960, 5,556,995)	−8452 (−11,588, −5461)	106.96 (58.79, 166.06)	Cost-saving	Dominated	10,590,934 (7,168,218, 14,090,156)	11,660,507 (7,981,475, 15,542,891)
PV 41 vaccines per day	1185 (442, 2148)	1005 (512, 1531)	24,036 (18,316, 30,122)	5,556,980 (5,556,960, 5,556,997)	−8790 (−12,059, −5780)	108.17 (60.17, 168.55)	Cost-saving	Dominated	10,952,992 (7,538,641, 14,709,589)	12,034,693 (8,324,080, 16,108,503)
**PV 54 vaccines per day**	**1167 (422, 2131)**	**976 (492, 1516)**	**24,000 (18,422, 30,080)**	**5,556,980 (5,556,960, 5,556,997)**	**−8825 (−12,340, −5528)**	**108.64 (60.8, 168.55)**	**Cost-saving**	**Lowest cost scenario**	**10,998,280 (7,376,307, 14,807,615)**	**12,084,723 (8,042,368, 16,253,473)**
Pre-emptive vaccination (PV) plus reactive vaccination (RV) at 135 doses per day when outbreak is triggered, otherwise at rate given below										
PV 27 vaccines per day	1216 (550, 2091)	1026 (604, 1493)	24,111 (18,207, 30,118)	5,556,979 (5,556,960, 5,556,996)	−8715 (−11,907, −5659)	107.48 (58.79, 168.4)	Cost-saving	Dominated	10,864,178 (7,348,999, 14,585,129)	11,938,965 (8,055,610, 15,932,332)
PV 41 vaccines per day	1173 (442, 2092)	978 (512, 1466)	24,001 (18,316, 29,829)	5,556,980 (5,556,959, 5,556,997)	−8825 (−12,263, −5797)	108.46 (60.51, 169.05)	Cost-saving	Dominated	10,993,629 (7,516,415, 14,960,349)	12,078,191 (8,310,368, 16,311,787)
PV 54 vaccines per day	1166 (422, 2063)	963 (492, 1435)	24,030 (18,422, 29,512)	5,556,980 (5,556,959, 5,556,997)	−8796 (−12,165, −5639)	108.84 (60.99, 169.27)	Cost-saving	Dominated	10,972,394 (7,275,536, 15,021,348)	12,060,798 (7,975,867, 16,358,494)

Not all vaccination scenarios are shown—these can be seen in Appendix Table S14b. Point values are means and ranges are 95% credibility intervals.

Cost-saving scenarios save money and gain QALYs compared to the no vaccination counterfactual scenario. Dominated scenarios have higher mean costs and save fewer QALYs (just comparison of mean) than another scenario. Extended dominated scenarios have a higher mean ICER and save fewer QALYs (just comparison of mean) than another scenario. The scenario in bold is the most cost-effective vaccination scenario—it saves most money and no other scenario that saves more QALYs is cost-effective compared to it. That is, the most CE scenario is pre-emptive vaccination at 54 doses per day when there is no outbreak and increased to 81 doses per day when there is an outbreak.

QALY, Quality Adjusted Life Years; ICER, incremental cost-effectiveness ratio; INMB, Incremental Net Monetary Benefit; PV, pre-emptive vaccination; RV, reactive vaccination.

### Baseline cost-effectiveness projections

Over the 10-year period, the counterfactual scenario projects 5,556,871 (95%CrI 5,556,794–5,556,936) discounted QALYs. All vaccination scenarios produce greater QALYs. For example, reactive vaccination at 135 vaccine doses per day gains 105 (95%CrI 54–168) QALYs compared to the counterfactual (Table 4).

The total discounted costs of the counterfactual scenario are £32,825,745 (95%CrI £25,193,861–39,213,245; Table 4) over 10 years, with clinical case management and public health responses costing £6,475,331 (20%) and £26,350,415 (80%), respectively (Appendix Table S14a). All vaccination scenarios decrease the total costs compared to the counterfactual (i.e., are cost-saving) because vaccination costs are offset by large savings in clinical care management and public health responses. The most cost-effective vaccination scenario (highest INMB; Table 4) is pre-emptive vaccination at 54 vaccine doses per day paired with reactive vaccination at 81 doses per day when an outbreak is triggered. This saves £8,825,393 (95%CrI 5,528,434–12,340,092) and gains 109 (95%CrI 61–169) QALYs compared to the counterfactual (Appendix Fig. S8 shows the cost-effectiveness plane for this scenario and four alternative vaccination scenarios). In the fully incremental analysis, all other vaccination scenarios are dominated, except for pre-emptive vaccination at 81 or 135 per day which both have ICERs above £30,000/QALY gained compared to this scenario.

### Threshold vaccine price

For pre-emptive vaccination at 54 vaccine doses per day, the threshold vaccine price for this strategy to be cost-effective is £325 or £305 per dose for the £20,000/QALY and £30,000/QALY WTP criteria, respectively (Appendix Table S16), whereas it is £313 and £290 for reactive vaccination at 81 vaccine doses per day, and £324 and £304 for the combined scenario. Across all vaccination rates considered, the highest threshold vaccine cost was £330 and £309 per dose for the £20,000/QALY and £30,000/QALY criteria, respectively, for pre-emptive vaccination at 27 doses per day. In general, threshold vaccine prices do not vary much by vaccination rate but are generally lower for reactive vaccination (versus pre-emptive vaccination at same vaccination rate; Appendix Fig. S9).

### Sensitivity analyses

The effect of the sensitivity analyses on the incremental costs and QALYs for each vaccination scenario are shown in Appendix Tables S15–S35 and described in Appendix pp 22–27, with the most cost-effective scenarios for each sensitivity analysis given in Table 3. These sensitivity analyses change the incremental costs and QALYs saved, but most do not majorly change which vaccination scenario is most cost-effective. Pre-emptive vaccination with reactive vaccination remains the most cost-effective scenario, but with a lower or the same pre-emptive vaccination rate (13–54 vaccine doses per day) and the same or higher reactive vaccination rate (81–204 vaccine doses per day) when we assume longer duration of vaccine protection, lower rates of imported infections, higher outbreak response criteria, use different data to define high-risk GBMSM, assume all high-risk GBMSM transition to low-risk before they age out of the model, allow revaccination for individuals with waned immunity, increase the time horizon to 20 years, or assume a shorter effective infectious period after 2024 (due to better symptom recognition). The most cost-effective approach becomes just pre-emptive vaccination at a higher rate (of 81 per day or more) when we include productively losses from mpox, or assume a lower discount rate, shorter duration of vaccine protection, higher rates of imported infections, lower outbreak response criteria, fractional doses are used, or change the distribution of time GBMSM remain within the modelled population from exponential to gamma distributed. Importantly, these sensitivity analyses suggest that the higher responsive vaccination rate done during outbreaks in the baseline scenario, or an even higher vaccination rate should be done continuously. Other sensitivity analyses have no effect on the preferred vaccination scenario, including assuming no risk reduction during outbreaks, breakthrough infections have less severe symptoms, or a greater proportion of GBMSM attending SHS. The only scenario where the most cost-effective approach becomes just reactive vaccination is when we don’t include public health response costs. Importantly, for this scenario and when we use different data to define high-risk GBMSM, the model suggests that the vaccine needs to be cheaper for vaccination to be cost-effective, with vaccination being cost-effective if the vaccine is less than £110 per dose.

## Discussion

Utilising an mpox transmission model, we evaluated the cost-effectiveness of different vaccination strategies for minimising future mpox outbreaks among GBMSM in England. Without future vaccination, our model projects mpox outbreaks will become more likely due to waning population-level immunity and despite responsive reductions in sexual risk behaviour during each outbreak. Conversely, if the vaccine has moderate cost (£160 per dose), we project that vaccinating for mpox is always better than not vaccinating; it reduces the number of cases, saves money and gains QALYs. Baseline projections suggest the most cost-effective strategy is routine pre-emptive vaccination of high-risk GBMSM at a moderate rate (54 doses per day) paired with reactive vaccination at a higher rate (81 doses per day) when an outbreak is triggered, translating to approximately 7300 vaccination doses being given each year. Vaccination remains cost-effective unless the vaccine cost is high (more than £330 per dose) and in specific sensitivity analyses described below.

To our knowledge, this is the first cost-effectiveness analysis of mpox vaccination. Strengths of the analysis include its use of cost data from the 2022 outbreak in England and the use of a validated model calibrated to this outbreak.^10^ However, uncertainties exist, and so extensive sensitivity analyses were undertaken to assess the robustness of our findings under alternative assumptions. As described below, these sensitivity analyses generally show the preferred strategy remains pre-emptive vaccination with a higher reactive vaccination rate or changes to just pre-emptive vaccination at a higher rate.

First, there is uncertainty around the vaccine’s duration of protection and whether individuals will be revaccinated or boosted in future. Our sensitivity analyses show our findings are generally robust to this uncertainty, with the preferred strategy remaining pre-emptive with reactive vaccination if we assume longer duration of protection or allow revaccination of individuals with waned immunity, but changing to pre-emptive vaccination if we assume shorter duration of protection. However, it is important to note that our cost-effectiveness projections allowing revaccination are likely to be optimistic because our model assumes that people become immediately eligible for vaccination once their protection wanes, which would be hard to determine in reality; this vaccination strategy needs to be considered more fully in future analyses. It is also uncertain what criteria will be used to trigger an mpox outbreak response, however, we found this did not majorly affect the preferred vaccination strategy, with a combined scenario either being preferred or it changing to pre-emptive vaccination at a higher rate.

Second, uncertainty exists in the importation rate of future mpox cases and their characteristics. Our sensitivity analyses show that if the importation rate increases, a high rate of pre-emptive vaccination may be the preferred strategy. Conversely, the preferred vaccination strategy remains unchanged if we assume a lower importation rate or mpox infections have a shorter effective infectious period from 2024 (due to increased symptom awareness). There is also uncertainty surrounding how the new mpox clade Ib may spread in the UK,^12^ with evidence suggesting sexual transmission^31^ and transmission among women and children being important.^31^ It is likely that a wider range of population groups will be vaccinated if there is an outbreak of mpox clade Ib in England; our model cannot evaluate such vaccination strategies and so they were not considered in this analysis.

Third, uncertainty exists in the sexual risk behaviour of high-risk GBMSM, and whether their risk behaviour reduces as they age or during future outbreaks.^11^ Our sensitivity analyses show that the preferred vaccination strategy remains unchanged when we use different data to define high-risk GBMSM, assume no risk reduction during future outbreaks or high-risk GBMSM gradually transition to being low-risk over time. However, when we use different data to define high-risk GBMSM, vaccination is now only cost-effective if the vaccine costs less than £145 per dose. This scenario assumes a larger group of high-risk GBMSM with lower transmission risk, which results in more GBMSM needing to be vaccinated to control the epidemic and less impact being achieved from vaccinating each GBMSM making it less cost-effective. Our model also assumes that individuals who have not attended sexual health services in the last 4 months are unlikely to get vaccinated. When we assumed a greater proportion of GBMSM could be vaccinated, the preferred vaccination strategy remained the same. Conversely, the preferred vaccination strategy changed to just pre-emptive vaccination (at a higher rate) when we modified the model to not assume a constant leaving rate for GBMSM, but instead assume the duration that GBMSM remain in the model is gamma distributed.

Fourth, uncertainty exists in the true costs of future outbreaks and choice of costs that should be included in economic evaluations. For instance, evidence suggests that the clinical features of mpox re-infections or breakthrough infections after vaccination are less pronounced than during the 2022 outbreak,^32^ and so their management costs could be less than we estimated. Our sensitivity analyses show that the preferred vaccination strategy remains unchanged when we assume breakthrough infections have half the chance of experiencing moderate and severe mpox disease. Conversely, the preferred strategy becomes just reactive vaccination if we do not include public health response costs for future outbreak, because less costs are saved from vaccination. In this scenario, the vaccine needs to cost less than £110 per dose for vaccination to be cost-effective. Alternatively, if we include mpox-related productivity losses then more costs are saved from vaccination, and the preferred vaccination strategy becomes pre-emptive vaccination (at a higher rate). Lastly, a 10-year frame was assumed in our cost-effectiveness analysis; the preferred vaccination strategy remained unchanged when a longer time frame was used (20-years).

Fifth, no HRQoL utility weights exist for mpox and so we used general utility weights for infectious diseases from the Global Burden of Disease study.^20^ To counter this issue, we included large uncertainty ranges around our utility estimates, and our projections were robust despite this. Our model also did not include worse health outcomes among people living with HIV^33^ because most GBMSM living with HIV in England are virally suppressed.^34^ This limits the generalisability of our model for settings where more people living with HIV are unsuppressed. In these settings, mpox vaccination could be more cost-effective because it reduces the severity of mpox disease among people with HIV.^35^

There are numerous existing model analyses considering the transmission of mpox. Model analyses of the mpox outbreak in 2022^9^^,^^10^^,^^36^ have suggested that the existing roll-out of vaccination has been important for preventing future resurgences, including for new clade I outbreaks.^37^ Other model analyses have suggested that future outbreaks could be controlled through vaccinating close contacts of cases^38^ or individuals with high numbers of sexual contacts,^39^ especially if done pre-emptively.^40^ Our analysis builds on these previous studies by evaluating the cost-effectiveness of future vaccination strategies.

In conclusion, our analysis shows that vaccination of high-risk GBMSM attending sexual health services is likely to be a cost-saving strategy for minimising future mpox outbreaks in England. These findings underpinned the evidence considered by the UK Joint Committee on Vaccination and Immunisation for recommending this vaccination strategy in England.^41^ Although projections suggested specific vaccination rates were optimal, these were uncertain and will be difficult to achieve in practise. Therefore, because most vaccination scenarios were cost-saving, we recommend that combined pre-emptive and reactive vaccination strategies should be implemented, with vaccine delivery rates being assessed as part of the planning for programme implementation, accounting for supply constraints, stakeholder priorities, and potential eligibility criteria. The robustness of our findings also suggests that similar vaccination strategies should be considered by other high-income countries for minimising mpox outbreaks going forward. This is particularly pertinent considering the continued circulation of mpox cases in these settings^12^ and the expanding outbreak in sub-Saharan Africa.^12^ In this region, the cost of the vaccine needs to be minimised to enable equitable access, which is crucial for achieving global control of mpox. Future studies need to determine the optimal vaccination strategies in such settings including for controlling new clade Ib infections.

## Contributors

PV, JGW, XSZ, SM and HM conceived and designed the analysis. XSZ developed the model and performed all model analyses. JGW and SN led and undertook the costing analysis and JGW oversaw the cost-effectiveness analysis. MM was involved with collection, collation and interpretation of cost data, and providing a link between the modelling team and the staff with access to the cost data. The cost data were extracted and prepared by ES, MK, TM, EJ, LG, SMo, DP and CD. SA, BD and BO extracted and prepared vaccination data while JIA provided case data. PV oversaw the overall analysis. XSZ wrote the initial draft of the manuscript with PV. All authors contributed to guiding the overall analysis plan, interpreting interim and final results, and critically reviewing the final version of the manuscript. XSZ, JGW and PV have directly accessed and verified the underlying data of this research article, and PV made the final decision to submit the manuscript.

## Data sharing statement

This analysis and modelling were undertaken for health protection purposes under permissions granted to UKHSA to collect and process confidential patient data under Regulation 3 of The Health Service (Control of Patient Information) Regulations 2020 and Section 251 of the National Health Service Act 2006. All data were pseudonymised during analysis, and records were stored securely. As such, authors cannot make the underlying datasets publicly available for ethical and legal reasons, particularly due to the sensitive information included. Applications for relevant anonymised data should be submitted to the UKHSA Office for Data Release at https://www.gov.uk/government/publications/accessing-ukhsa-protected-data. The model code and projections for this paper will be shared with interested parties upon reasonable request, which will be decided by Peter Vickerman, Josephine Walker and Xu-Sheng Zhang.

## Declaration of interests

PV and JGW have received unrestricted research grants from Gilead not related to the submitted work and PV has received consulting fees off GSK not related to this work. SN received grant funding off Pfizer unrelated to this work. HM is a member of multiple guideline writing committees for the British Association for Sexual Health and HIV. This research was funded in whole, or in part, by the National Institute for Health Research Health Protection Unit for Behavioural Science and Evaluation at the University of Bristol [NIHR200877]. This research was also funded in part by the Health Protection Research Unit on Bloodborne Viruses and Sexually Transmitted Infections at University College London.
